# Exploring and Targeting the Tumor Immune Microenvironment of Neuroblastoma

**DOI:** 10.33696/immunology.3.111

**Published:** 2021

**Authors:** Katherine E. Masih, Jun S. Wei, David Milewski, Javed Khan

**Affiliations:** 1 Oncogenomics Section, Genetics Branch, Center for Cancer Research, National Cancer Institute, NIH, Bethesda, MD, USA; 2 Cancer Research UK Cambridge Institute, University of Cambridge, Cambridge, England

## Abstract

Pediatric neuroblastoma is a heterogenous disease that accounts for significant morbidity and mortality in children. Deep genomic and transcriptomic profiling of patient tumors has revealed a low mutational burden and a paucity of therapeutic targets. Furthermore, different molecular subtypes, such as *MYCN* amplification, have been associated with adverse outcomes. Using whole transcriptome sequencing, we previously explored the immune microenvironment of neuroblastoma subtypes and discovered its association with clinical outcome. Specifically, we found that patients with tumors infiltrated by higher levels of cytotoxic lymphocytes had a better overall survival. Additionally, we found that a high *MYCN* gene expression signature in *MYCN*-non-amplified tumors is an independent predictor of adverse outcome. However, signatures of tumor infiltrating cytotoxic immune cells in this subtype of tumors predict an improved outcome. While this is clinically informative, it does not provide a full picture of the dynamics underlying the biology of tumor immune microenvironment and how to use this information to improve patient outcomes. Here, we highlight our previous work and current approaches using immunotherapy in neuroblastoma and explore our current understanding of the immune biology of these tumors. We further describe how this correlates with patient outcome, and how this information can be used to develop novel immunotherapeutic strategies for pediatric patients with neuroblastoma.

## Introduction

Neuroblastoma is derived from the developing sympathetic nervous system and is the most common extracranial solid tumor of childhood. It is typically diagnosed within the first two years of life and accounts for approximately 15% of all pediatric cancer deaths [1,2]. The clinical courses of these patients vary drastically, from spontaneous regression of low-risk stage 4S tumors in infants with no therapeutic interventions to continued disease progression of high-risk tumors despite aggressive chemotherapy, radiotherapy, and surgical interventions [3,4]. Unfortunately, the cure rate for high-risk neuroblastoma, defined as *MYCN-*amplified (*MYCN*-A) tumors or a diagnosis over the age of 18 months with metastatic disease, remains lower than 50%. Additionally, there is significant morbidity associated with the aggressive multimodal therapies used in this patient population. Therefore, the development of targeted therapies with lower toxicities is imperative.

Large-scale sequencing efforts have revealed a low overall mutational burden and very few recurrently mutated genes in neuroblastoma, which makes devising targeted therapies difficult [5–7]. More recently, immunotherapeutic approaches, utilizing monoclonal antibodies targeting GD2, combined with IL-2, GM-CSF, and isotretinoin, have demonstrated improved survival in the high-risk patient population [8,9]. Unfortunately, this approach is associated with significant toxicities, and 40% of these patients still relapse, suggesting a need for a deeper understanding of the underlying biology of the tumor cells and their interaction with the tumor microenvironment (TME) including immune and stromal cells. Studies from our group and others have utilized RNA-seq to reveal correlations between the tumor-infiltrating immune cells and clinical characteristics in patients with neuroblastoma. Tumor-associated macrophages (TAMs) are known to have an increased frequency in metastatic disease and are associated with a decreased progression free survival [10]. Conversely, our group has demonstrated that a subset of high-risk *MYCN*-not-amplified (*MYCN*NA) neuroblastomas have increased T-cell infiltration, suggesting the presence of antigenic targets and an active anti-tumor immune response [11,12]. We further showed in two independent patient cohorts that a high *MYCN* functional gene expression signature in *MYCN*-NA tumors is associated with decreased immune infiltrate and a poor overall survival. In this commentary, we review previous studies to interrogate the TME, highlight the diverse TME subtypes of neuroblastoma, and explain why profiling the TME is clinically relevant. We further propose future research directions and therapeutic opportunities informed by studies of the TME to ultimately improve patient outcomes.

### RNA Profiling to Assess the TME

Historically, methods such as flow cytometry or immunohistochemistry have been used to understand the composition of the TME, but limitations such as sample quantity, requirement of viable cells, and insufficient number of pre-selected markers have prevented the accurate identification of infiltrating cells among a large number of tumor samples [13]. Therefore, much of the initial knowledge about the TME across patient samples has been discovered using bulk RNA profiling followed by various *in silico* methods to infer cell composition in tumor samples. The two major categories of computational approaches to understand heterogenous cellular components with mRNA gene expression data are marker gene-based or deconvolution. Marker gene-based methods use predefined gene sets that are characteristic of specific cell types. Enrichment scores are calculated relative to where these specific marker genes reside within the expression-ranked gene list to estimate the abundance of cell types within a tumor sample. Conversely, deconvolution methods model the gene expression data of a sample as the weighted sum of its heterogenous cell populations [14]. Within the overarching category of deconvolution approaches, multiple methods, such as ESTIMATE, CIBERSORT, and cytolytic scores have been developed [15–18]. ESTIMATE provides relative abundance of tumor, stromal, and immune cells, while CIBERSORT can be used to estimate the relative abundance of 22 immune cell types within a tumor sample [15,17,19]. Additional clarity can come from calculating cytolytic scores based on mRNA expression of genes such as granzymes and perforin, which correlate to T- or NK-cell cytolytic activity in a tumor [18]. The primary benefit of these bulk mRNA profiling methods over an antibody approach to detect protein levels, such as flow cytometry or imaging methods, is that they enable marker-agnostic discovery from a single assay. However, they cannot provide precise cellular quantification, heterogeneity, and spatial resolution of single cells within a tumor sample.

### Profiling Cytotoxic T-cells Infiltrating Neuroblastomas

**Figure 1:**
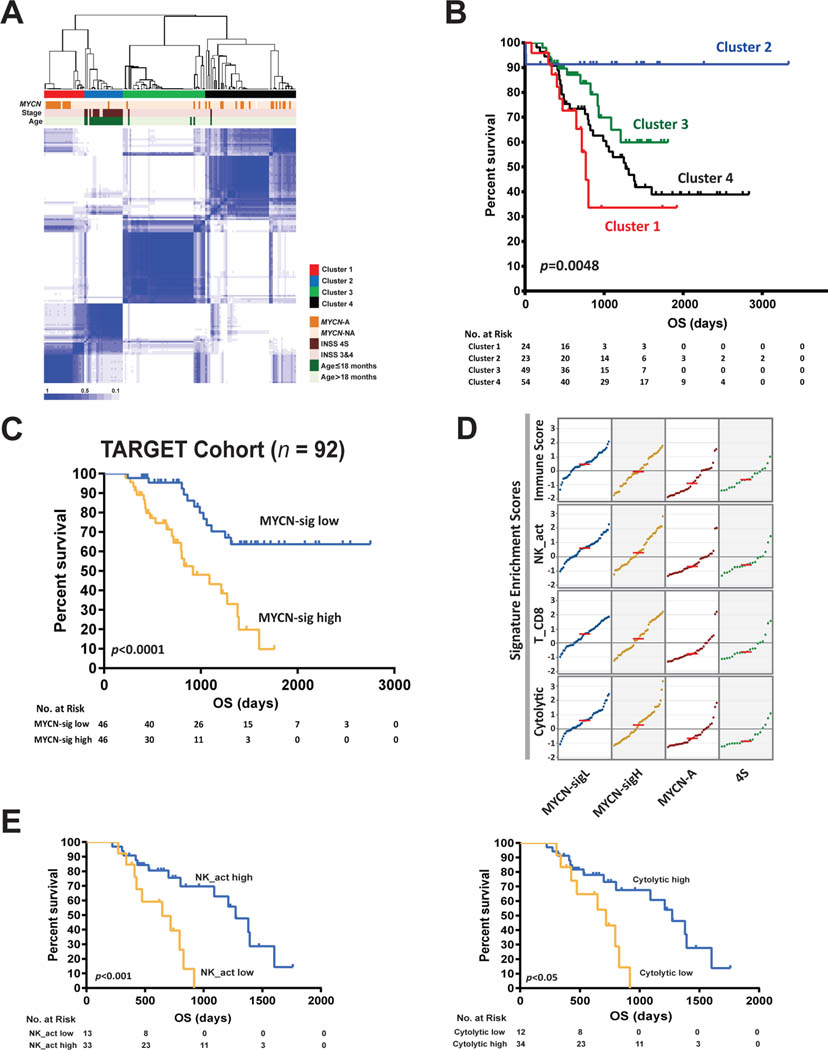
TME composition is associated with tumor subtype and patient clinical outcomes. **A.** Consensus clustering of 150 TARGET neuroblastoma samples based on their gene expression profiles identified 4 distinct clusters. **B.** Kaplan–Meier curves demonstrated significant difference in overall survival (OS) in all patients for the 4 clusters identified by consensus clustering in **A**. **C.** The *MYCN*-functional gene signature predicted the outcome of 92 TARGET high-risk patients with *MYCN*-NA neuroblastomas. Median *MYCN*-sig score was used to stratify the *MYCN*-NA patients into two groups used in the Kaplan–Meier plot. A significantly worse OS was observed for patients with a high *MYCN*-signature (*MYCN*-sig high) than for those with low *MYCN*-signature (*MYCN*-sig low). **D.** Single sample GSEA (ssGSEA) showed *MYCN*-NA tumors had higher immune scores, activated NK cell, CD8 T-cell, and cytolytic activity than those of *MYCN*-A or 4S tumors. Enrichment scores were z-scored, sorted, and plotted for each group of tumors. The red lines are median values for each plot. **E.** Kaplan-Meier survival analysis of high-risk TARGET patients with *MYCN*-NA tumors expressing a high *MYCN*-signature stratified by the scores of the activated NK cell or cytolytic signatures. The survival curves were obtained using a K-M optimization procedure, and samples with expression levels of activated NK cell or cytolytic signatures less than or equal to the optimal threshold were labeled as “NK_act low” or “Cytolytic low”. Samples with the expression level of the signatures greater than the threshold were labeled as “NK_act high” or “Cytolytic high”.

We initially reported a study using deep transcriptomics to profile neuroblastomas from pediatric patients as part of an NCI TARGET (Therapeutically Applicable Research to Generate Effective Treatments) cohort [6,12,20]. We performed RNA-seq on 150 pre-treatment, clinically annotated, patient neuroblastomas consisting of mostly high-risk tumors with a goal of uncovering the tumor intrinsic and extrinsic biology underlying this disease and its relation to clinical outcomes. We did not find an association between mutational burden and the quantity of tumor infiltrating lymphocytes. An unsupervised, consensus clustering approach identified four groups with unique molecular signatures, clinical characteristics, and survival probabilities (Figure 1A) [21]. Cluster 1 was defined by *MYCN*-A tumors, while Cluster 3 and Cluster 4 were primarily composed of high-risk *MYCN* -NA tumors. All three of these clusters were associated with poor overall survival. Alternatively, Cluster 2 was mainly comprised of stage 4S tumors from younger patients and was associated with better outcomes (Figure 1B). We discovered that a functional *MYCN* gene signature (*n*=157 genes) is an independent predictor of poor outcome for patients with *MYCN*-NA tumors [22]. We demonstrated this in both the TARGET cohort (*n*=92) and an independent cohort of high-risk patients with neuroblastoma (*n*=150) (Figure 1C) [16,22].

**Figure 2:**
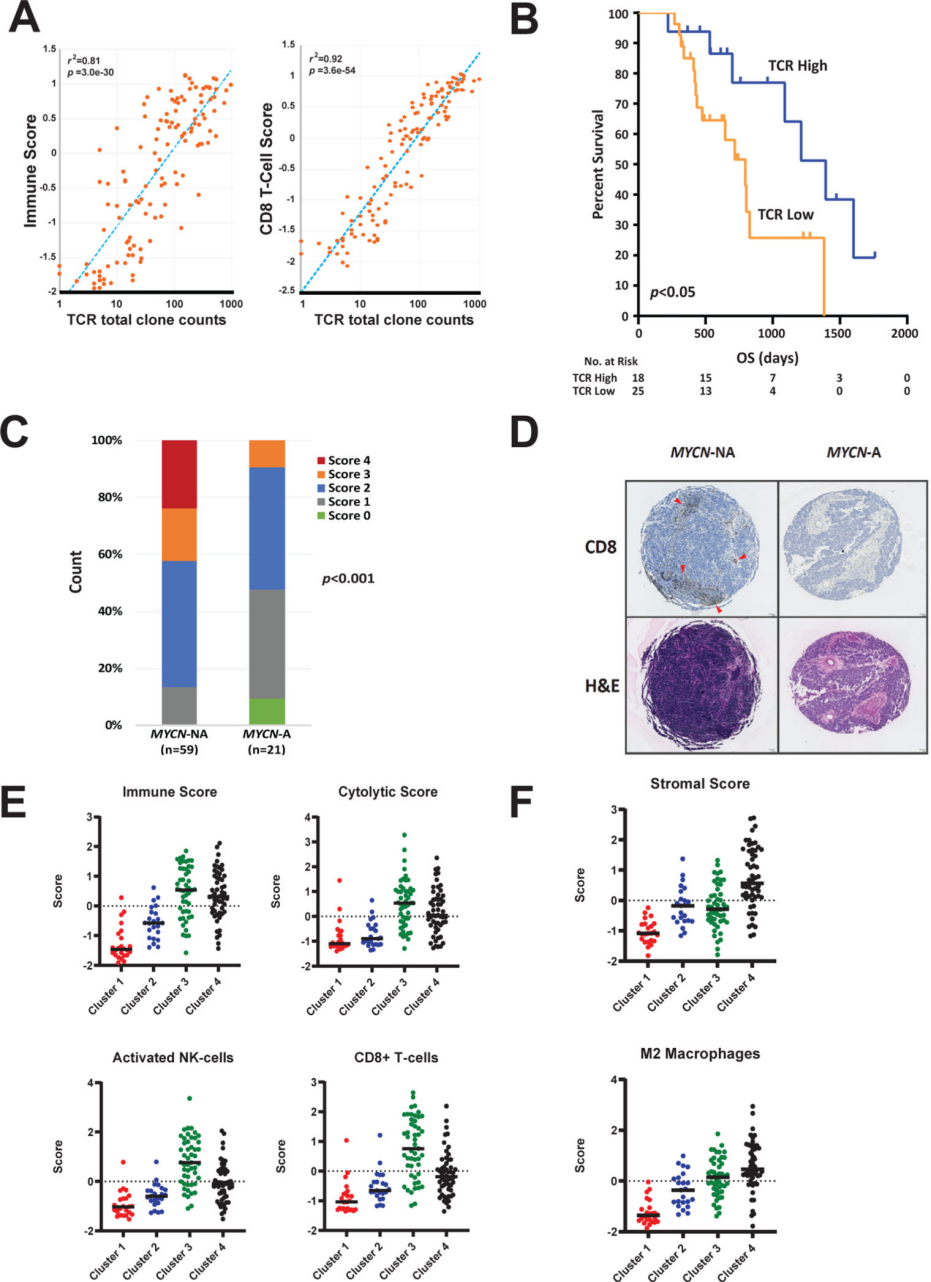
*MYCN*-amplification and expression levels of the functional *MYCN* gene signature influences immune microenvironment infiltrate **A.** TCR total clone count is significantly and highly correlated with the immune score and CD8 T-cell score for the TARGET cohort, indicating a major contribution of cytotoxic T-cells to the immune signatures detected in neuroblastoma samples. **B.** TCR total clone number is significantly associated with the outcome for patients with *MYCN*-NA neuroblastoma expressing a high *MYCN*-signature. **C.** Color bars depict the count percentage for CD8 scores across tumor types. **D.** IHC from two representative *MYCN*-A or *MYCN*-NA neuroblastomas. Red arrows point out multiple aggregates of CD8+ T cells in a *MYCN*-NA tumor, which are absent in the *MYCN*-A neuroblastoma. **E.** Anti-tumor CIBERSORT scores (z-scored normalized) for each sample separated by cluster demonstrates an increased in anti-tumor immune infiltrate, including CD8+ T-cells and activated NK-cells in a subset of *MYCN*-NA tumors. **F.** Pro-tumor CIBORSORT scores (z-scored normalized) for each sample separated by cluster demonstrates a relatively immunosuppressive microenvironment through an increased M2 macrophage and stromal scores in another subset of *MYCN*-NA (Cluster 4).

We then evaluated the clusters’ relative immune scores and found that the *MYCN*-NA tumors had significantly higher scores compared to *MYCN*-A tumors (Figure 1D). Subsequently, we found that 6 out of 22 immune signatures were significantly associated with prognosis in patients with *MYCN*-NA tumors expressing a high *MYCN* functional gene signature, where a higher immune signature was associated with improved outcome [12]. Of these, activated NK-cell, CD8^+^ T-cell, and cytolytic signatures were the most significantly different between clusters (Figure 1D). Among them, high NK-cell and cytolytic signatures predicted outcome within *MYCN*NA tumors with high *MYCN* gene signatures (Figure 1E). Cytolytic signatures were highly correlated with NK-, T-, and B-cell signatures. This suggested that the presence of activated cytotoxic immune cells is prognostic in high-risk patients with *MYCN*-NA tumors expressing a high *MYCN* functional gene signature. Thus, we hypothesized that there would be an increase in T-cell receptor (TCR) clones recognizing tumor specific antigens. We found that the number of total TCR clones was correlated with both the CD8^+^ T-cell score and overall immune scores across all samples (Figure 2A). In concordance with immune scores, the total TCR clone counts were not only higher for *MYCN-*NA tumors but independently associated with outcomes in patients with *MYCN*-NA tumors expressing a high *MYCN-*signature (Figure 2B). These findings were independently validated using immunohistochemistry for CD8^+^ T-cells in 80 patient neuroblastoma samples, which showed that the increased CD8^+^ T-cell scores are significantly higher in *MYCN*-NA tumors compared to *MYCN*-A tumors (Figures 2C and 2D). This is consistent with previous reports that *MYCN* and *MYC* can downregulate the expression of HLA molecules, which are crucial for antigen presentation on the cell surface to cytotoxic T-cells [23–25].

T- and NK-cell exhaustion can allow tumors to evade killing by the patient’s immune system [26,27]. In order to better characterize the phenotype of infiltrating T-cells, we examined co-expression of a panel of 7 exhaustion marker genes in combination with the cytotoxic immune cell signatures in a subset of *MYCN*-NA neuroblastoma tumors and observed a positive correlation between immune exhaustion and cytotoxic activity [12]. Interestingly, two of the exhaustion markers, *CTLA4* and *PDCD1* (PD1), are targets of FDA approved therapeutics, which may have the potential to improve outcomes in this high-risk neuroblastoma patient population.

In summary, our findings of prognostic immune signatures including cytotoxic (T and NK) cell activation, expansion of TCR clones, cytolytic signatures, upregulation of immunosuppressive markers, indicates that the TILs may be capable of specifically targeting neuroblastoma cells. This may be harnessed for combinatorial immunotherapeutic approaches, which could improve patient outcomes. Overall, our study demonstrates a strong correlation between the neuroblastoma TME and patient outcome.

### Uncovered Subtypes of Neuroblastoma TMEs

**Figure 3:**
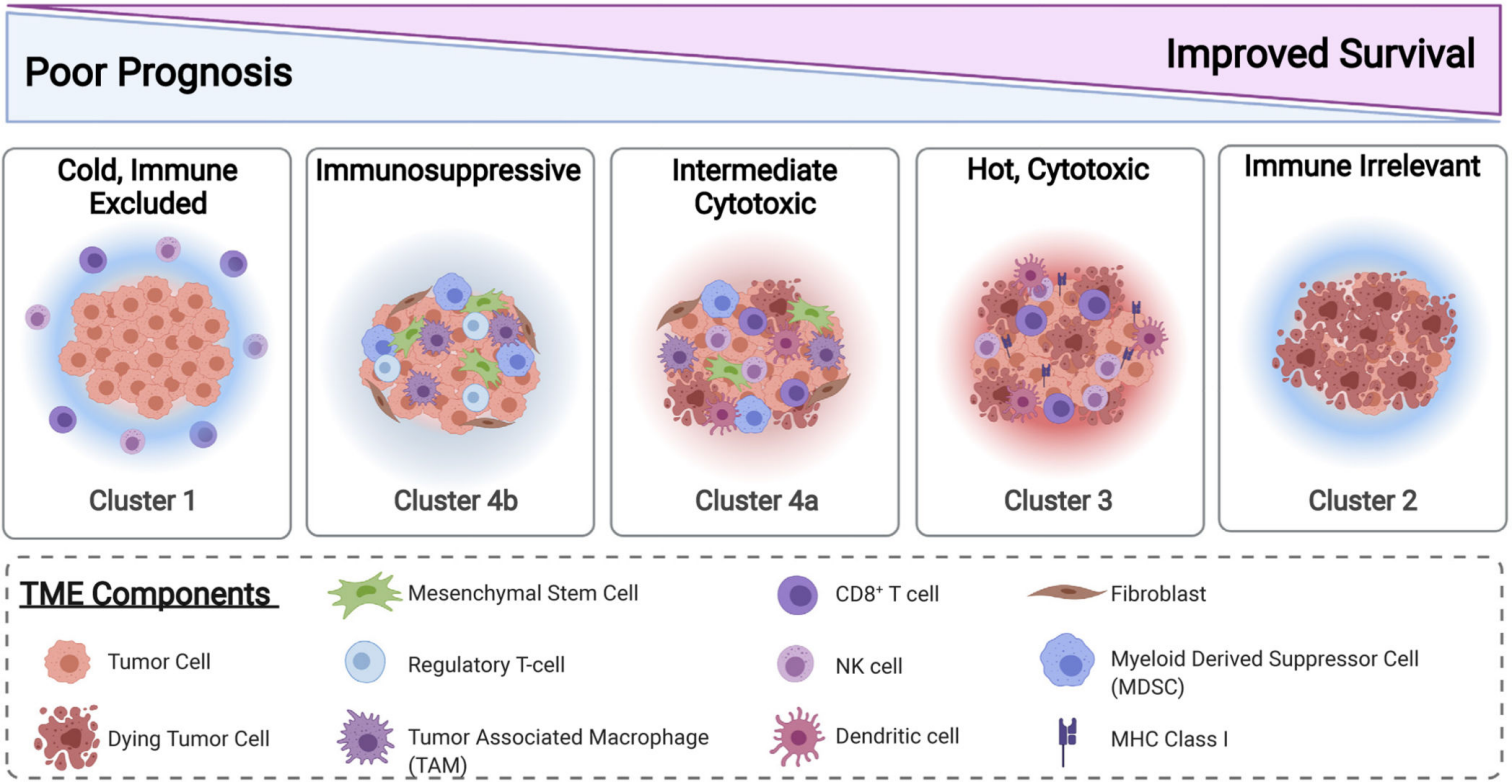
Proposed TME subtypes predicted by transcriptomics.

Our data support that the TME of neuroblastoma is dynamic, influenced by tumor molecular phenotype, and correlated with patient outcomes. Cluster 1 tumors are from ultra-high-risk patients with *MYCN*-A tumors, which are characterized by a “cold” TME with few immune cell infiltrates (Table 1 and Figures 2C–F and 3). It has previously been shown that surface expression of MHC Class I is downregulated in *MYCN*-A tumors through epigenetic silencing [23–25]. A decrease in MHC Class I could provide an explanation for the relatively lower abundance of tumor infiltrating CD8^+^ T-cells in *MYCN*-A neuroblastoma.

Cluster 2 tumors were from patients who are low-risk infants, less than 18 months of age with 4S tumors. These tumors generally regress spontaneously with minimal therapy despite a relatively cold TME (Table 1 and Figures 2 and 3). Because of this, we regard these TMEs as “immune irrelevant”; therefore, incorporating immunotherapies into the existing therapeutic approach is not necessary as it is unlikely to improve outcomes for this group of patients.

Cluster 3 tumors are primarily *MYCN*-NA tumors with a “hot” TME. Although patients with these tumors are clinically high-risk, they have elevated infiltrating anti-tumor immune cells, such as NK-cells and CD8^+^ T-cells and higher immune cytotoxic activity reflected by cytolytic scores, in contrast to Cluster 1 tumors (Table 1; Figure 2E–2F). This is associated with a significantly improved clinical outcome. Interestingly, we also saw higher expression of immune checkpoints in *MYCN*-NA tumors with increased CD8^+^ T-cell infiltrates, which is supported by previous findings that signaling from T-cells can upregulate immune checkpoint expression by tumor cells [28]. It is likely that these variations in TME composition are due to interactions between immune cell subsets and the tumor cells themselves. Given our data supporting that these tumors have a cytotoxic TME, it is possible that patients with TMEs characteristic of Cluster 3 prior to treatment may be primed to respond to immunotherapy approaches such as anti-GD2 or checkpoint blockade immune therapies (Figure 2E).

In contrast to Clusters 1–3, we observed an increased stromal signature in Cluster 4 of which tumors are predominantly *MYCN*-NA (Table 1; Figure 2F and 3). Patients with tumors in this cluster are also stratified as high-risk and have a worse outcome compared to those falling into Cluster 3 (Figure 1B). This is consistent with previous studies demonstrating that pro-tumor cells, such as regulatory T-cells, TAMs, such as myeloid derived suppressive cells (MDSCs), and stromal cells, such as cancer associated fibroblasts, contribute to an immunosuppressive microenvironment in neuroblastoma [10,29]. Cluster 4 can be further subdivided based on infiltration of anti-tumor immune cells (Cluster 4a) or infiltration of solely pro-tumor immunosuppressive and stromal cells (Cluster 4b) (Table 1 and Figure 3).

Overall, our data demonstrate that *MYCN*-A tumors overwhelmingly have cold, immune excluded microenvironments. However, in the case of *MYCN*-NA tumors, multiple TME subtypes may exist. As response to immunotherapies remains inconsistent across patients with high-risk neuroblastoma, it is likely that these differences lie in the tumors’ TMEs. Therefore, profiling the TMEs of *MYCN*-NA tumors may identify biomarkers to improve risk stratification and development of novel treatment approaches for patients with high-risk neuroblastoma.

### Current Immunotherapeutic Approaches for Neuroblastoma

The twenty-first century has brought with it a push to identify more targeted therapies with improved side effect profiles for patients with cancer, and one major area of development is utilizing the patient’s immune system with immunotherapies. For pediatric patients with neuroblastoma, out of the 590 total clinical trials registered on ClinicalTrials.gov, 86 of these utilize an immunotherapeutic approach (Tables 2 and S1). The most established approach in this patient population involves using monoclonal antibodies (mAbs) targeting GD2, a tumor associated antigen (TAA), which is uniformly overexpressed on neuroblastomas relative to normal tissues [30–32]. Although these approaches have improved outcomes in some patients with high-risk neuroblastoma [33,34], this response is not uniform, and currently, there are not reliable biomarkers to predict clinical response to the anti-GD2 therapy.

Immune checkpoint blockade (ICB), which utilizes mAbs targeting molecules known to dampen anti-tumor immune responses, has shown dramatic results for adult patients with solid tumors, and agents targeting the immune checkpoints CTLA-4 and PD-1 have been approved by the FDA [35]. Ipilimumab was the first FDA approved mAb targeting CTLA-4, and it has been approved to treat melanoma [36,37], in addition to subtypes of renal cell carcinoma and colorectal cancer in combination with the PD-1 inhibitor nivolumab [38,39]. Nivolumab, and other inhibitors of the PD-1/PDL-1 signaling, can also be used independently to treat multiple cancer types, including melanoma, multiple types of lung cancer, Hodgkin’s lymphoma, hepatocellular carcinoma, and urothelial cancers [35,40–42]. There have been clinical trials aimed at recapitulating these results in pediatric solid tumors, including neuroblastoma (Table 2 and Table S1). Unfortunately, the results have been disappointing with very few patient responses to therapy. It is possible that ICB can be useful in enhancing responses to other targeted therapies, and there is an ongoing UK-based phase I clinical trial combining nivolumab with an antibody-drug conjugate targeting GD2 (NCT02914405).

More recently, adoptive cell therapies (ACTs) have been trialed to treat neuroblastoma. The majority of these are using CAR T-cells to target surface antigens such as GD2, CD171, CD276, or EGFR, and there are a total of 18 completed or ongoing CAR T-cell trials treating neuroblastoma patients (Table 2 and Table S1) [43–46]. Unfortunately, the response rates seen in these trials are not as promising as those seen in pediatric patients with leukemia [47–49]. It is thought that heterogenous expression of TAAs, hostile TMEs, and reduced persistence of CAR T-cells are all contributing to the lack of clinical responses in patients with solid tumors.

### Outlook of Immunotherapy for Pediatric Neuroblastoma

Our group and others have shown that there are different subtypes of TMEs found in neuroblastoma and that the TME of pediatric neuroblastoma is not only associated but plays an active role in the clinical outcomes of these patients [10,12,50,51]. A variety of therapeutic approaches to harness a patient’s immune system to target the tumor have been developed. However, responses to these individual treatments are inconsistent and overall clinical trial results have been unsatisfactory. Furthermore, it has been established that TMEs are dynamic, particularly after exposure to conventional therapies [52–54]. Therefore, we propose that profiling of individual patient’s TMEs has the potential to guide clinical decision making, and rational, combinatorial approaches of these immunotherapeutic strategies may improve patient outcomes.

We hypothesize that response to mAbs targeting TAAs in neuroblastoma may be predicted by the presence of cytotoxic cells, such as NK-cells and T-cells in the TME, such as those seen in Cluster 3 and Cluster 4a. There are several mechanisms of action of mAbs, but many are dependent on cytotoxic innate or adaptive immune cells. For example, they can directly activate complement mediated cytotoxicity or their effects can be indirect through antibody-dependent cellular cytotoxicity (ADCC) [55]. When GD2 on a neuroblastoma cell is targeted by the mAb, it is first recognized and opsonized by binding of the antibody’s Fc region mediated by the Fcγ receptor (CD16) on NK-cells [56]. This recognition triggers ADCC when the NK-cells release cytotoxic granules. While NK-cells are the main effectors of ADCC, myeloid cells such as monocytes, macrophages, neutrophils, and dendritic cells have also been implicated as effectors [57]. Once cell death is initiated, other intracellular TAAs can be processed and presented by antigen presenting cells, directing the adaptive immune system against the tumor, in a process known as epitope spreading [58].

Many neuroblastomas lack tumor infiltrating lymphocytes (TILs) in their TMEs, such as tumors categorized as Cluster 1 or Cluster 4b. One of the causes can be attributed to low immunogenicity of tumor subtypes. It has been shown that increased expression of MYCN through amplification or its functional gene signature can downregulate MHC Class I on the cell surface, which cells use to present antigens to T-cells [23–25]. Strategies, such as the use of hypomethylating agents or interferon-γ or IL-15 tethered to immune cells, could help upregulate MHC and render these tumors responsive to immunotherapeutic approaches [59–62]. Alternatively, adoptive cell therapies that function independently of MHC, such as CAR T- or NK-cells targeting highly expressed surface antigens could be attractive options for tumors with lower immunogenicity.

One established mechanism of resistance to ACT is immune cell exhaustion [63–65]. Exhaustion has proven to be especially relevant in ACT approaches against solid tumors, and several strategies have been employed to circumvent this phenomenon. T-cell intrinsic editing, such as overexpression of c-Jun or using a 4–1BB costimulatory domain have alleviated exhaustion and increased CAR T-cell persistence [66,67]. Additionally, our data shows an association between increased T-cell infiltration and expression of immune checkpoints, such as CTLA-4 and PD-1 [12]. Therefore, it is rational that utilizing combined ICB targeting multiple checkpoints, particularly in tumors falling into Cluster 3 or Cluster 4a could potentially be effective. Alternatively, ICB combined with cellular immunotherapies targeting TAAs could increase therapeutic efficacy [68–70].

Besides cytotoxic immune cells, other components of the TME, such as cytokines and immunosuppressive cells, have been associated with clinical outcomes and identified as sources for sub-optimal ACT activity. Therefore, modulating the immunosuppressive elements of the TME could improve therapeutic responses. TGF-β has been implicated in suppressing cytotoxicity and infiltration of both NK-cells and T-cells [71–73]. Targeting TGF-β signaling either by tyrosine kinase inhibitors or genetically engineering dominant negative TGF-β receptors into CAR T-cells have shown improved T-cell proliferation and improved tumor killing in other solid tumors [74,75]. Given the presence of both cytotoxic and immunosuppressive cells in Cluster 4a, this approach could be efficacious in this tumor type. Alternatively, therapies designed to target tumor cells may also exert pleiotropic effects on the TME. For example, all trans retinoic acid, a common drug to treat high-risk neuroblastoma, suppresses myeloid derived suppressive cells (MDSCs) in the TME of sarcomas [76]. More recently, genetically engineered myeloid cells (GEMys) expressing IL-12 have been shown to traffic to immunosuppressive TMEs and downregulate myelosuppressive macrophages and promote cytotoxic T-cell activity [77]. These approaches could be particularly relevant to Cluster 4 neuroblastoma TME subtypes with high MDSC infiltration.

## Conclusions

Neuroblastoma displays four recurrent patterns in the composition of the TME. These patterns are associated with distinct patient outcomes and may indicate tumor susceptibilities to specific immunotherapies. Currently, predicting responses to immunotherapies remains challenging, and since their mechanisms of action are dependent on the interplay between a patient’s tumor and immune system, understanding the TMEs of individual tumors could provide clinical rationale for selecting therapeutic approaches. Furthermore, as the TME can evolve over time under therapeutic pressures, evaluation of these changes in the context of exposure to therapy to better understand therapeutic effects on the TME and to optimize the timing and choice of cancer treatments including immunotherapeutic strategies for these patients.

In summary, neuroblastoma transcriptome profiling has revealed notable characteristics of the TME including immune infiltrates in high-risk neuroblastoma and their relationship to patient outcome. These results warrant further studies, including spatial transcriptomics or proteomics to validate and develop a deeper understanding of the interplay between tumor cells and TME. Only with this knowledge, biomarkers based on tumor and TME phenotypes can then be identified to guide patient stratification and therapeutic strategies to improve the outcomes for patients with high-risk neuroblastoma.

## Supplementary Material

JCI-21-112_Supplementary file

## Figures and Tables

**Table 1:** Clinical relevance of TME characterization of neuroblastoma based on RNA-seq.

Cluster	Risk/Outcome	TME Characterization	*MYCN* Amplification	Pro-Tumor TME Elements	Anti-Tumor TME Elements
1	Ultra-high-risk/Very Poor	Cold, immune excluded	*MYCN*-A >>> *MYCN*-NA	Low MHC	Low infiltrate
2	Low-risk/Favorable	Immune irrelevant (Stage 4S)	*MYCN*-NA	N/A	N/A
3	High-risk/Moderate	Hot, cytotoxic	*MYCN*-NA >> *MYCN*-A	Low suppressive and stromal; immune checkpoints	High infiltrate, high MHC
4a	High-risk/Poor	Intermediate cytotoxic	*MYCN*-NA >> *MYCN*-A	MDSCs, high stromal; immune checkpoints	Higher than 4b
4b	High-risk/Poor	Immunosuppressive	*MYCN*-NA >> *MYCN*-A	MDSCs, high stromal	Lower than 4a

**Table 2:** Summary of clinical trials using immunotherapy in neuroblastoma.

Category	Target	Total Trials	Completed	Active (Not Recruiting)	Recruiting
**BiTE+ACT**	GD2	1	*N/A*	*N/A*	*N/A*
**CAR T-cell**	CD171	1	*N/A*	*N/A*	1
	CD276	2	*N/A*	*N/A*	1
	CD276, GD2, PSMA	1	*N/A*	*N/A*	1
	EGFR	1	*N/A*	*N/A*	1
	GD2	10	2	1	5
	GD2 (+ virus specific T-cells)	3	1	2	*N/A*
**ICB**	CTLA-4	2	2	*N/A*	*N/A*
	CTLA-4 and PD-1	1	*N/A*	*N/A*	1
	PD-1	4	*N/A*	1	*N/A*
**ICB + mAb**	PD-1 + GD2	1	*N/A*	*N/A*	1
**mAb/ADC**	EGFR	1	1	*N/A*	*N/A*
	GD2	46	20	11	5
	GD2 + CD47	1	*N/A*	*N/A*	*N/A*
	IGF-1	1	1	*N/A*	*N/A*
	NGNA	3	1	*N/A*	1
	TNFa	1	*N/A*	*N/A*	*N/A*
**NK-CAR**	GD2	1	*N/A*	*N/A*	*N/A*
**TIL**	non-specific tumor	1	*N/A*	*N/A*	*N/A*
**Vaccine**	CTAs	2	*N/A*	1	*N/A*
	Tumor specific	2	2	*N/A*	*N/A*

BiTE: Bispecific T-cell Engager; ACT: Adoptive Cell Therapy; ICB: Immune Checkpoint Blockade; mAb: Monoclonal Antibody; ADC: Antibody-drug Conjugate; TIL: Expanded Tumor Infiltrating Lymphocytes
